# Rewired Metabolism of Amino Acids and Its Roles in Glioma Pathology

**DOI:** 10.3390/metabo12100918

**Published:** 2022-09-28

**Authors:** Sirui Chen, Jingjing Jiang, Ao Shen, Ying Miao, Yunfeng Cao, Ying Zhang, Peiyu Cong, Peng Gao

**Affiliations:** 1 College of Medical Laboratory, Dalian Medical University, Dalian 116044, China; 2 Clinical Laboratory, The Second Hospital of Dalian Medical University, Dalian 116023, China; 3 Department of Anesthesiology, Shengjing Hospital of China Medical University, Shenyang 110004, China; 4 HE University, Shenyang 110163, China; 5 E&M College, Shenyang Aerospace University, Shenyang 110136, China; 6 Shanghai Institute for Biomedical and Pharmaceutical Technologies, Shanghai 200237, China; 7 Internal Medicine Department, Dalian Public Health Clinical Center, Dalian 116033, China; 8 Neurosurgery Department, Affiliated Dalian Municipal Central Hospital of Dalian Medical University, Dalian 116022, China

**Keywords:** amino acid, glioma, metabolomics, metabolism, biomarker

## Abstract

Amino acids (AAs) are indispensable building blocks of diverse bio-macromolecules as well as functional regulators for various metabolic processes. The fact that cancer cells live with a voracious appetite for specific AAs has been widely recognized. Glioma is one of the most lethal malignancies occurring in the central nervous system. The reprogrammed metabolism of AAs benefits glioma proliferation, signal transduction, epigenetic modification, and stress tolerance. Metabolic alteration of specific AAs also contributes to glioma immune escape and chemoresistance. For clinical consideration, fluctuations in the concentrations of AAs observed in specific body fluids provides opportunities to develop new diagnosis and prognosis markers. This review aimed at providing an extra dimension to understanding glioma pathology with respect to the rewired AA metabolism. A deep insight into the relevant fields will help to pave a new way for new therapeutic target identification and valuable biomarker development.

## 1. Introduction

Amino acids (AAs) are the exclusive building blocks for proteins in both the eukaryotes and the prokaryotes. It is estimated that over 500 AAs could be found in different forms of lives [1], but only 20 AAs were implicated in protein synthesis. The proteogenic AAs share a similar chemical structure with a carboxyl group and an amino group, together with a hydrogen atom, bonded to the α-carbon atom. The physical and chemical properties of different AAs are determined by the relevant side chain groups which are also covalently linked to the α-carbon atom. Owning to the chirality of the α-carbon atom, each AA has two optical isomers in theory. Interestingly, the 20 proteogenic AAs are all L-enantiomers. Previously, it was believed that AAs of D-enantiomers were absent in mammals. However, mounting evidence demonstrated that D-AAs could also be found in higher animals. For example, D-serine and D-aspartate were abundant in the central nervous system (CNS) of some vertebrates [2,3]. This review would pay more attention to L-AAs.

Except for protein synthesis, AAs have diverse functions under different circumstances [4]: (a) Some AAs could be converted to corresponding α-ketonic acids by aminotransferases. These α- ketonic acids could be catalyzed to glucose, fats or ketone bodies to be utilized for various purposes. The relevant AAs are called ketogenic or glycogenic AAs. (b) Some AAs behave as neurotransmitters, precursors for many hormones or other bioactive molecules. For example, glutamate is an excitatory AA in the CNS [5]. One of the enzymatic products of glutamate, the γ-aminobutyric acid, is an inhibitory neurotransmitter [6]. (c) Glycine, serine, methionine and histidine could provide one-carbon units, promoting purine, pyrimidine, and the redox regulator biosynthesis [7,8]. (d) Some AAs have been utilized as potential biomarkers to aid diagnosis and prognosis. For instance, elevated circulating branched-chain AAs (BCAAs) indicate a higher risk of pancreatic cancer [9]. Individuals with elevated serum concentrations of 9 AAs showed a higher incidence of Type 2 diabetes [10]. (e) AAs could behave as epigenetic modifiers to regulate cell phenotypes. Sarcosine played roles in prostate cancer diagnosis and stratification [11,12], and this AA also brought about increased methylated CpG island landscapes in several prostate cancer cell lines [13]. Collectively, AAs are multi-functional molecules.

Metabolites directly affect and regulate cellular phenotypes [14,15]. Cell transformation (tumorigenesis) is accompanied by metabolic reprogramming [16]. A half-century ago, Warburg published his profound work pointing out that tumor cells consumed a large part of glucose through glycolysis even under the circumstance of sufficient oxygen supply [17,18]. Several lines of evidence demonstrated that metabolic reprogramming is involved in diverse metabolic pathways when the cell transformed [19].

Glioma is one of the lethal malignancies in the CNS and accounts for one-third of the primary brain tumors [20]. The World Health Organization primarily stratified gliomas into four histopathologic grades. The higher the grade, the less favorable the prognosis [21]. The exact pathological mechanisms of gliomas are still elusive. A deep insight into the metabolic adaptation of gliomas is of benefit to exploring new therapeutic solutions. This review would shed light on the rewired metabolism of AAs and its contribution to glioma’s malignant behaviors. Additionally, the diagnostic and prognostic values of AAs are introduced briefly.

## 2. Glioma AA Metabolism Adapted to Proliferation

One of the hallmarks of cancer cell metabolism is their uncontrollable proliferation abilities. This does not mean the malignant cells adopted some metabolic pathways that were unique to tumor cells. Most frequently, cancer cells change certain enzymes’ expression or activities to meet their abnormal catabolic and anabolic requirements [19]. The relevant mechanisms include the mutations of specific enzyme genes, the accumulation of specific activators/inhibitors, the altered post-translational modification activities and the over-activated/inhibited regulation signals.

**Figure 1:**
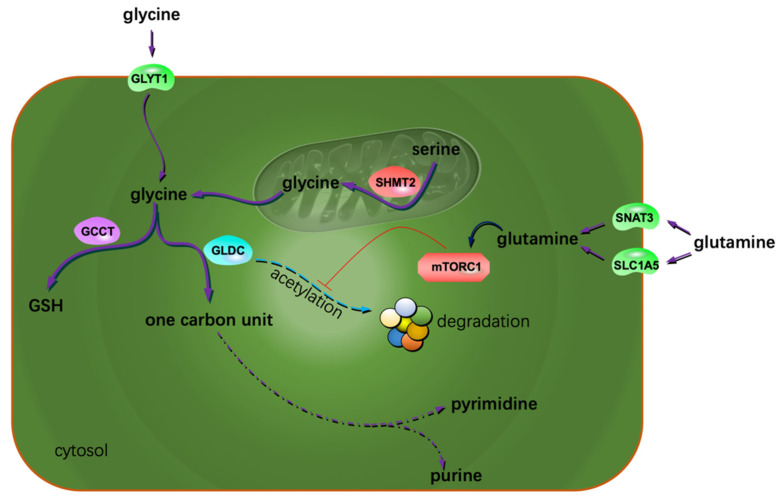
Extra glycine and glutamine sustain glioma proliferation. The possible mechanism is that both the AAs contribute to the metabolism of one carbon units and the reduction substances. The arrow with a blunt end indicates suppression.

One-carbon unit metabolism is closely linked to many biological processes. The production of ATP, NADPH, lipids and nucleotides greatly relies on one-carbon units, especially the de novo synthesis of purine. Cell proliferation is largely determined by the availability of nucleotides [22]. Jain, M et al. profiled the extracellular consumption and release of 219 metabolites for 60 primary human cancer cell lines including U251 glioblastoma (GBM) cells [23,24]. They found glycine was consumed massively by rapidly proliferating cells and released by slowly proliferating ones. The uptake of extracellular glycine is mediated by the transporter of GLYT1 [25]. The intracellular biosynthesis of glycine could occur both in the cytosol and the mitochondria. Most cells default to the mitochondria for glycine synthesis [26]. Mitochondrial serine hydroxymethyltransferase 2 (SHMT2) catalyzes the reversible transformation from glycine to serine [27]. SHMT2 was frequently found to be overexpressed in GBM tissues [28,29]. Isotope tracing analysis indicated mitochondria contribute about two-thirds of the needed glycine to rapidly proliferating glioma cells [24]. The consumed glycine was either converted to one-carbon units to support purine synthesis or incorporated into glutathione (GSH) to clear reactive oxygen species (ROS) [30,31]. Glycine decarboxylase (GLDC) plays a key role in converting glycine into one-carbon units. The activity of GLDC is regulated by acetylation modification. This post-translational modification of GLDC is inhibited by the mechanistic target of rapamycin complex 1 (mTORC1). Acetylated GLDC is prone to be degraded in the proteasomes and results in impaired pyrimidine synthesis and growth inhibition of gliomas [32]. Many glioma cells exhibit highly expressed mTORC1 and GLDC [32,33]. γ-Glutamylcyclotransferase (GGCT), one of the key enzymes promoting GSH synthesis, was demonstrated to be highly expressed in glioma cells. Suppression or depletion of GGCT compromised glioma cells but not normal cells’ proliferation [34,35], implying the different GSH-related vulnerabilities of the normal and the transformed cells. In addition to GGCT, SHMT2 also promoted GSH synthesis [26]. Collectively, glycine confers gliomas a proliferative advantage by providing one-carbon units and reduction substances (Figure 1). The voracious appetite for glycine of gliomas renders glycine a promising imaging tracer to aid glioma aggressiveness evaluation [36]. Additionally, one-carbon metabolism has been identified as one of the potential targets for treating GBM [37].

Many cancer cells greatly rely on glutamine to sustain proliferation [38,39,40,41]. Most of the tumor cells depend on aerobic glycolysis for energy production. This results in limited carbon sources entering the tricarboxylic acid (TCA) cycle. The rapidly proliferating cells need many precursors originating from the TCA cycle for building block generation. To this end, glutamine is directed to the TCA cycle to replenish the cellular carbon pool in the form of glutamate [42]. Glioma cells are glutamine-addicted both for biosynthetic and energetic purposes [43]. The increased glutamine uptake is ascribed to the highly expressed glutamine transporters. There are at least four glutamine transporters in mammalian cells. Glioma cells greatly rely on the transporters of SLC1A5 and SNAT3 to take up glutamine [44,45]. This feature constitutes the basis of glutamine-dependent imaging technology for glioma diagnosis and tumor boundary delineating [46,47,48]. The entry of glutamine sometimes activates mTORC1 [45], facilitating glioma cell proliferation, in part through the mechanisms described above (Figure 1).

**Figure 2:**
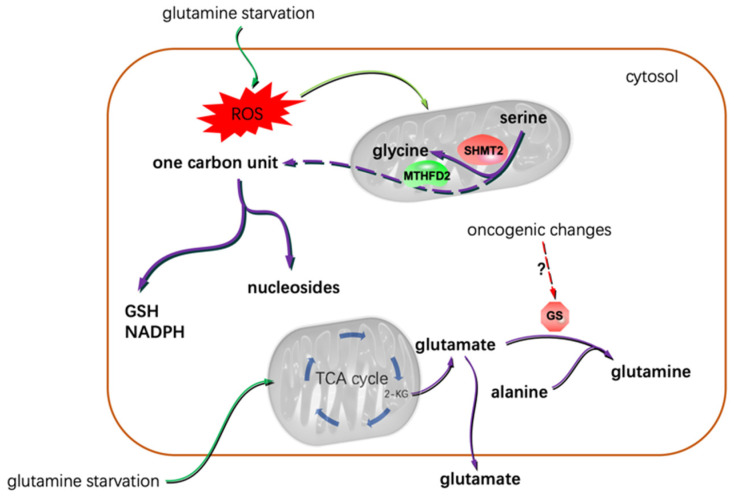
Glutamine starvation resulted in the dependence of glioma cells on serine-based one carbon unit metabolism, and that gliomas showed differential glutamine addiction was most possibly due to the varied oncogenic backgrounds of the cells. Under glutamine deprivation conditions, some glioma cells even excreted glutamate [49]. 2-KG: α-ketoglutarate.

Gliomas’ glutamine addiction implies that the intrinsic synthesis capacity of glutamine is beyond glioma’s metabolic need. Whereas, Tardito, S. et al. reported that not all glioma cell lines are sensitive to glutamine starvation. In some GBM cell lines, glutamine consumption and the degree of cellular glutamine dependency exhibited no correlation [49]. Neither glutamine starvation nor glutaminase inhibition conspicuously affect cell growth. Specifically, insufficient glucose-based carbon sources from the TCA cycle to glutamate-dependent glutamine synthesis inhibited proliferation [49]. Under glutamine starvation conditions, glutamine is not directed to the TCA cycle to replenish cellular carbon sources in the form of glutamate. On the contrary, together with the alanine-originated nitrogen, the TCA cycle provides glucose-based carbon sources for glutamate production (Figure 2). The glutamine synthetase (GS) catalyzes the reaction from glutamate to glutamine. Under glutamine starvation conditions, it is the GS-dependent glutamine synthesis that fuels the de novo intracellular purine biosynthesis.

GS is differentially expressed by glioma cells [49]. Gliomas harbor many genetic mutations, which might differentially affect the cell’s fate under glutamine starvation conditions [50,51,52]. For example, glutamine withdrawal from the media resulted in 80% growth inhibition of the LN18 cells. The inhibition rate of the U251 cells was three times higher than that of the LN18 cells [49]. About 50% of the GBM tissues carry EGFR mutations. EGFR could promote glioma cell proliferation through glutamate dehydrogenase-1-dependent glutaminolysis [53]. The isocitrate dehydrogenase (IDH) mutations frequently occur in gliomas and confer gliomas an extra dependency on glutamine to survive or proliferate [54]. Evidently, as of proliferation, oncogenic changes delimit glioma metabolic adaptation to some extent.

Excessive ROS are frequently generated under hypoxic conditions. Gliomas outgrow their oxygen supply. This hypoxic stress impairs intracellular redox homeostasis. Under this circumstance, increased GSH synthesis is crucial for the cells to survive [55]. Glutamine deprivation not only triggers endogenous glutamine synthesis but also brought about redox stress [56,57]. Glioma cells cultured under glutamine starvation conditions showed upregulated expression of SHMT2 and methylenetetrahydrofolate dehydrogenase 2 (MTHFD2) [58]. Both of the enzymes participate in mitochondrial one-carbon unit metabolism. Generally, MTHFD2 expression responds to a more oxidative mitochondrial redox potential [59]. Knockdown of MTHFD2 in U87 and T98 GBM cells under glutamine-deprived conditions led to elevated cytotoxicity due to abnormal oxidation status and excessive ROS generation. Under this circumstance, serine-dependent one-carbon unit metabolism provided reducing power by promoting NADPH and GSH synthesis (Figure 2) [60]. This could be corroborated by the findings that the central areas of the resected GBM tissues (prone to suffering from hypoxia) exhibit extremely high serine and glycine content compared to the adjacent normal tissues [58]. Thus, the increased serine and glycine in the center areas of the GBM tissues indicates an active response to the requirement of both nucleoside synthesis and reducing power production (Figure 2).

The metabolic effects of AAs could work synergistically. Cystine depletion could induce ferroptosis [61]. Methionine deprivation resulted in cell cycle arrest [62]. When the two AAs were depleted simultaneously, glioma cells suffered from additionally increased ROS and decreased GSH levels [63]. The double deprivation also resulted in autophagy. This synergistical effect opens up a new window to seek therapeutic solutions by manipulating multiple metabolic pathways simultaneously.

## 3. Glioma Migration, Invasion, and AA Metabolism

Although the metastasis of glioma is seldom found in patients, the infiltrative growth feature of gliomas is very harmful. Thus, understanding the mechanisms of glioma invasion and migration would benefit patient care.

**Figure 3:**
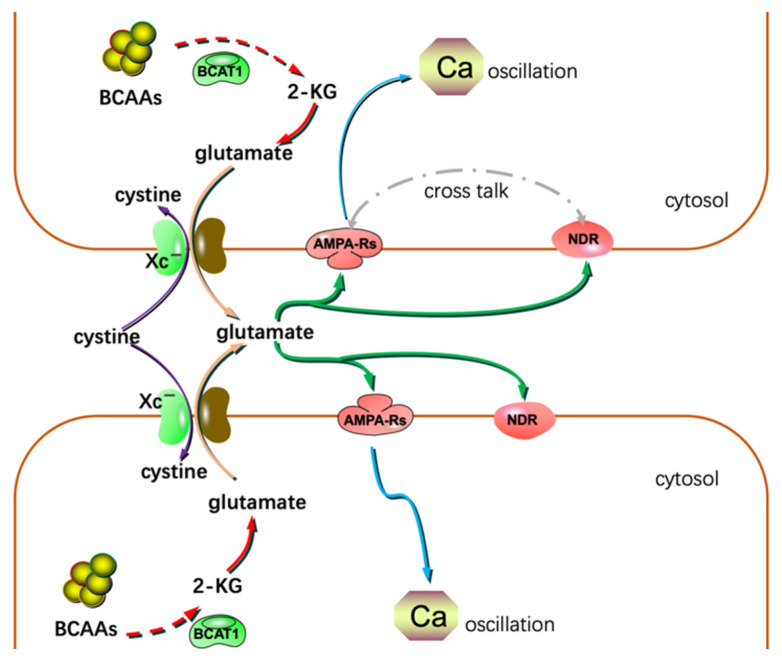
Glutamate could act as a hormone to stimulate invasion and migration in addition to its cytotoxic effects. NDR: N-methyl-D-aspartate receptor; 2-KG: α-ketoglutarate.

System Xc^−^ is an Na^+^-independent antiporter. It mediates the exchanging of extracellular cystine and intracellular glutamate [64]. Most gliomas highly express system Xc^−^. Glutamate excreted by gliomas is cytotoxic to the adjacent normal brain cells. Through this, gliomas create extra space to expand and invade. Lyons S. et al. reported that glutamate could activate Ca_2_^+^-permeable α-amino-3-hydroxy-5-methylisoxazole-4-propionic acid receptors (AMPA-Rs) in the manner of autocrine and/or paracrine signaling. Activation of AMPA-Rs resulted in intracellular Ca_2_^+^ oscillation, one of the essential signals to trigger glioma invasion and migration (Figure 3). Most of the glioma tissues highly express AMPA-Rs. Agonists of AMPA-Rs facilitated glioma invasion and migration [65]. Additionally, glutamate showed a high affinity to N-methyl-D-aspartate receptors. Crosstalk between AMPA-Rs and N-methyl-D-aspartate receptors has been demonstrated to synergistically promote glioma invasion, especially in a glutamate-rich microenvironment. BCAA transaminase 1 (BCAT1) initiates the breakdown of BCAAs. Some catabolic products of BCAAs could be used as the carbon skeleton for glutamate synthesis. Inhibition of BCAT1 resulted in a decreased efflux of glutamate and brought about the impaired invasiveness of glioma cells [66]. In conclusion, except for its cytotoxic effects, glutamate could act as a hormone to stimulate invasion and migration (Figure 3) [67].

Many tumors show arginine dependence, and some tumors are deficient in arginine synthesis [68,69]. Arginine deprivation resulted in an elongated cell shape and the absence of intracellular lamellipodium, resulting in cell motility, adhesion, and invasion suppression [70]. Arginylation is crucial for actin assembly. Arginine deprivation in GBM resulted in decreased N-terminal arginylation of β-actin and brought about impaired cell motility, adhesion and invasiveness abilities [70]. Sulforaphane–cysteine, a metabolite from sulforaphane, is abundant in broccoli. It could inhibit glioma cell migration and invasion by regulating mitophagy and the expression of invasion-related molecules [71].

Factors contributing to glioma proliferation often benefit migration and invasion. For example, glutamine depletion suppressed glioma growth, invasion, and migration [72]. N-acetylcysteine is an intermediate metabolite of GSH synthesis. It could decrease the expression of Notch 2 and the relevant downstream genes, resulting in proliferation, migration, and invasion suppression. Potentially, N-acetylcysteine might induce apoptosis [73]. Thus, any intervention affecting glioma proliferation might affect invasion and migration, and vice versa.

## 4. IDH Mutations and AA Metabolic Alteration

Gliomas harbor varied mutations and the relevant topics have been discussed widely [50,51,52,74]. Cancer-related mutations frequently occur in the genes whose protein products are transcriptional regulators, epigenetic modifiers, and signal transducers. Identifying specific mutations benefits glioma stratification and targeted therapies [75,76,77,78]. This section only pays attention to the mutations of a subclass of concrete metabolic enzymes, the IDHs.

IDH mutations are the only reported metabolic enzyme mutations that occur in gliomas. Mutations of IDH1 and IDH2 were found in >70% of lower-grade gliomas and some IDH-mutated high-grade gliomas [79,80]. Whereas, mutant IDHs were seldom found in the primary GBM patients [81]. These mutations perturbate many AAs’ metabolism.

Brain cells are the limited types of cells that could metabolize BCAAs [82]. The first catabolic step of BCAAs is the transposition of the α-amino group to α-ketoglutarate (2-KG). Two transaminases could catalyze the transamination reaction. One is the cytosolic BCAT1 and the other is the mitochondrial BCAT2 [83,84,85]. The expression of BCAT2 is ubiquitous and the BCAT1 is expressed only in limited tissues [86]. GBMs carrying wild-type IDH1 showed highly expressed BCAT1 [66]. IDH1 promotes cytosolic 2-KG production [87]. Supplying 2-KG to GBM cell cultures increased BCAT1 expression. Knockdown of *IDH1* led to downregulated BCAT1 but could be reversed by exogenous 2-KG [66]. 2-KG is an indispensable cofactor for 2-KG-dependent dioxygenases (2-KDDs). The mutated IDHs could convert isocitrate to 2-KG and then reduce 2-KG to α-hydorxyglutarate (2-HG) using NADPH [88,89]. Owing to their similar chemical structures [90,91], 2-KG and 2-HG could competitively bind to 2-KDDs. Many 2-KDDs are involved in DNA methylation modification. Gliomas with IDH mutations synthesize more 2-HG, which could inhibit 2-KDDs and result in DNA hypermethylation [86]. Ectopically expressed mutant IDHs could downregulate BCAT1 expression due to hypermethylation of the BCAT1 promoter. Gliomas release a large amount of glutamate to their adjacent tissues for expansion purposes. Inhibiting glioma BCAT1 expression reduces glutamate release with BCAAs’ accumulation. The accumulated BCAAs are not catabolized to generate acetyl-CoA effectively, resulting in impaired lipogenesis. Thus, highly expressed BCAT1 indicates a poor prognosis for gliomas with wild-type IDHs [92]. 2-HG could inhibit the activity of both BCAT1 and BCAT2, resulting in impaired synthesis of glutamate and the extensive reliance on glutaminase of gliomas [93]. Additionally, insufficient glutamate reduced GSH synthesis, contributing to glioma chemosensitivity [94]. Lower 2-KG levels resulted in less activated 2-KDDs and caused DNA hypermethylation, “mimicking” the IDH-mutated phenotypes [86]. From this aspect, IDH-mutated gliomas might benefit from 2-HG’s epigenetic modifications for proliferation, whereas, gliomas with wild-type IDHs might turn to BCAA catabolism to sustain effective proliferation.

The IDH1 gain-of-function mutations result in the reduction of 2-KG to 2-HG at the expense of NADPH. This helps to maintain a lower ratio of NADPH/NADP [95,96,97,98,99]. Nevertheless, a lower NADH/NAD ratio is also crucial to cancer cells [100]. Proline could be synthesized by NADH-dependent pyrroline 5-carboxylate reductase 1/2 (PYCR1/2) in the mitochondria using glutamine as a substrate [101]. Gliomas carrying IDH1 mutations employ a PYCR1-driven proline synthesis pathway to maintain a lower NADH/NAD ratio [101,102]. This activated PYCR1 pathway also partially uncouples the TCA cycle from respiration, benefiting some oxygen-independent biosynthesis processes e.g., citrate and aspartate generation. Given that gliomas often suffer from hypoxia, activating this proline synthesis pathway contributes to maintaining intracellular anabolic precursor homeostasis [102].

IDHs catalyze one of the important reactions in the TCA cycle. This cycle provides most of the carbon sources for non-essential AA synthesis. Imaginably, the mutant IDHs affect many non-essential AA metabolism. For example, after analyzing 224 different grades of glioma tissues, Bjorkblom et al. found higher levels of glycine and 2-aminoadipic acid in IDH mutated higher-grade gliomas. In low-grade astrocytoma and oligodendroglioma, elevated N-acetyl aspartic acid was closely related to IDH mutations [103]. In the cerebrospinal fluid from patients with IDH mutated gliomas, alanine was usually decreased [104]. Sometimes, IDH1 and IDH2 mutations could differentially affect the AA metabolism. For instance, under hypoxic conditions, decreased reductive glutamine metabolism was only found in gliomas with IDH1 mutations but not IDH2 mutations [105]. One of the possible reasons might be the different subcellular localization of IDH1 and IDH2.

## 5. Epigenetic Regulation Exerted by AA Metabolism

DNA methylation, nucleosome remodeling, histone modifications, and RNA-mediated regulations could alter gene expression patterns without changing the sequence of DNA. The relevant mechanisms are named epigenetic regulation or modification [106,107]. Epigenetic regulation plays a crucial role in tumorigenesis. The 2-KDD enzyme family includes many members and many of them take part in epigenetic modifications [108]. 2-KDDs utilize 2-KG and molecular oxygen as substrates and produce succinate and carbon dioxide. A structural analog of 2-KG could competitively inhibit 2-KDDs. The most studied competitor is 2-HG, one of the enzymatic products of the mutant IDHs.

**Figure 4:**
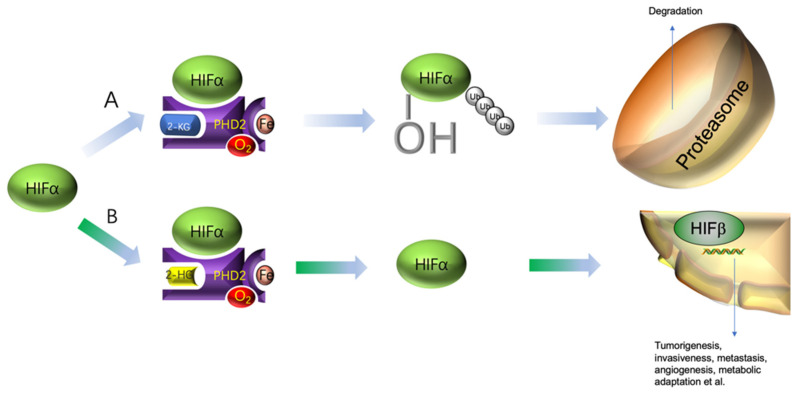
HIFα function and degradation. (**A**) Under normoxic conditions, HIFα will be hydroxylated by PHD2 and then subjected to ubiquitylation for degradation in the proteasome. (**B**) In the presence of some competitive inhibitors, e.g., 2-HG, HIFα will not be hydroxylated. The intact HIFα will bind to HIFβ and enter the nucleus to initiate the expression of many tumorigenesis genes.

Hypoxia-inducible factor-1 (HIF-1) is a transcription factor responsible for hypoxia adaptation. It consists oftwo2 subunits, HIF-1α and HIF-1β. HIF-1α has a short half-life of about 5 min and its degradation is oxygen-availability-dependent. Under normoxic conditions, HIF-1α is hydroxylated by prolyl hydroxylase domain-2 (PHD2), a representative member of the 2-KDDs. The hydroxylated HIF-1α is then degraded in the proteasome, resulting in nearly undetectable HIF-1α protein in the cytosol. Under hypoxic conditions, due to the lack of sufficient oxygen, PHD2 cannot properly hydroxylate HIF-1α. This intact subunit would translocate to the nucleus, dimerize with HIF-1β and become transcriptionally active. HIF-1 promotes many genes’ expression [109,110], and most of the activated genes facilitate tumorigenesis (Figure 4) [111,112]. Expression of HIF-1β is constitutive in mammal cells and the stability of HIF-1β was not affected by oxygen [110].

**Figure 5:**
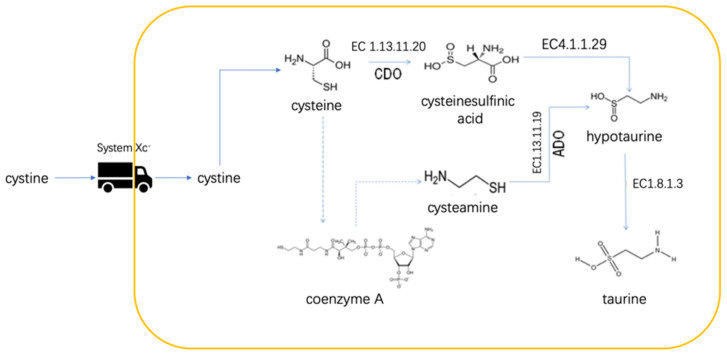
Schematic representation of biosynthesis of hypotaurine. The system Xc^−^ transports cystine into the cell. Cystine is catalyzed to cystine and then utilized by either cysteamine (2-aminoethanethiol) dioxygenase (ADO) or cysteine dioxygenase (CDO) for hypotaurine synthesis.

A previous study reported that hypotaurine, a nonprotein AA, was increased in glioma tissues. The tissue content of hypotaurine was tightly related to the tumor grades [113]. Hypotaurine promotes glioma cell proliferation and invasion concomitantly. Molecular docking indicated that hypotaurine could compete off 2-KG to bind to PHD2. The binding free energy of hypotaurine is lower than that of 2-KG and 2-HG. Hypotaurine could inhibit the hydroxylation of HIF-1α in a dose-dependent manner. Not limited to that, some other 2-KDDs involved in DNA methylation are also sensitive to hypotaurine inhibition [114]. The inhibition could be rescued by 2-KG in a dose-dependent manner. Challenged by hypotaurine, C6 glioma cells showed condensation of HIF-1 in the nucleus [114]. Hypotaurine could be synthesized by two distinct pathways (Figure 5) [115,116,117]. One way involves cysteine dioxygenase. Another way is the oxidation of cysteamine by cysteamine (2-aminoethanethiol) dioxygenase (ADO). In gliomas, the ADO pathway is very active [118]. Most of the needed cysteine is transported by the system Xc^−^ in the form of cystine [119]. Immunohistochemical staining showed that glioma tissues highly expressed system Xc^−^ especially in the higher-grade tumors [113]. Inhibiting system Xc^−^ resulted in decreased intracellular cysteine and, in turn, hypotaurine concentrations. Taurine is the oxidative product of hypotaurine. The intracellular concentration of taurine is not affected by hypotaurine and cysteine availability [113]. However, the ingestion of taurine by nude mice bearing U87MG xenografts could arrest the tumor growth, implying that taurine suppresses hypotaurine synthesis through negative feedback (Figure 5) [113]. Presumably, hypotaurine might confer gliomas a hypermethylation phenotype as 2-HG [120,121]. Unfortunately, there was no report about the relationship between hypotaurine and 2-HG. That hypotaurine and 2-HG could work separately or coordinately is worthy of exploring.

Except for IDH1 and IDH2, a third IDH paralog—IDH3—had been identified in eukaryotic mitochondria [122]. IDH3 catalyzes the reaction from isocitrate to 2-KG irreversibly and IDH3 is seldom mutated in gliomas [123]. May J. et al. reported that the expression of IDH3α was upregulated in GBM tissues [124]. Inactivation of IDH3α resulted in blunted one-carbon metabolism and abnormal epigenetic modifications. The cytoplasmic IDH3α could help to recruit cytoplasmic SHMT to the nucleus to provoke one-carbon unit metabolism [125]. In the presence of IDH3α, the availability of one-carbon units is dependent on serine. When IDH3α was silenced, one-carbon units were produced by the methionine salvage pathway. The IDH3α-dependent one-carbon units are involved in the epigenetic silence of many growth factors. IDH3α triggers the differential expression of genes related to methylation status. The mechanisms are unknown. However, IDH3α deficiency resulted in decreased 2-KG and increased succinate and fumarate [124]. Succinate and fumarate could inhibit 2-KDDs by employing a similar mechanism as that of 2-HG [90,126]. The hypermethylation phenotype might be ascribed to the effects of the two short-chain C4 organic acids. Presumably, crosstalk between AA metabolism and the TCA cycle cooperatively contributes to epigenetic modifications.

## 6. AA Metabolism and Glioma Chemotherapy Sensitivities

Many attempts have been made to potentiate cancer treatment outcomes in the light of interfering with tumor metabolism. The pilot trials were the prescriptions of folate antagonists for treating leukemia [127]. The recent decades have witnessed encouraging progress in the relevant fields, e.g., the advocation of the ketogenic diet [128,129]. Temozolomide (TMZ) was one of the first-line oral agents for glioma treatment. It can cause DNA methylation damage by depleting DNA methyltransferase, one of the repairing mechanisms of DNA impairment [130]. Asparaginase inhibition led to a reduced percentage of S-phase cells, augmented autophagy, and increased mitochondrial pathway-dependent apoptosis [131,132]. Depletion of asparagine by asparaginase with co-administration of TMZ could potentiate chemotherapy effects [133].

Cisplatin was one of the adjuvant reagents for glioma chemotherapy [134]. It can form adducts with cellular DNA and lead to cell-cycle arrest and apoptosis [135]. GSH can covalently bind to cisplatin to detoxify it. Depletion of GSH results in elevated intracellular cisplatin-DNA adducts and potentiated cisplatin cytotoxicity. The mechanism is that GSH deficiency rendered intact cisplatin more opportunity to reach DNA. The extra DNA damage also benefited TMZ therapy [136]. IDH1-mutated cells exhibit increased oxidative stress and are more sensitive to chemotherapy than their counterparts carrying wild-type IDHs [137]. Evidence from clinical practice demonstrated that interfering with GSH synthesis was a promising strategy adjuvant to various chemotherapy for gliomas with mutant IDHs [138]. When compared to the edge tissues of gliomas, the core tissues contain more tyrosine, and tyrosine aminotransferase is highly expressed in the core tissues. Additionally, most of the patients with activated tyrosine synthesis show poor prognoses. Based on these findings, Yamashita D. et al. postulated that tyrosine metabolism might affect chemosensitivities [139]. The exact mechanism was not well addressed.

Cell lines contain minority clones that possess stemness [140]. Immanuel S. et al. separated the neurospheroidal cells from the epithelial U87MG (eU87MG) population. They found that the neurospheroidal cells showed less sensitivity to TMZ [141]. The two cell populations exhibited a significant difference in tryptophan, glycine, alanine glutamine, proline, and serine consumption paradigms [141]. The stem cells were not as metabolically active as the ordinary cells. The dormant behavior was closely linked to their chemoresistance [142,143]. However, the specific AA metabolic profile of the stem cells should not be neglected.

## 7. AA Metabolism and Glioma Immune Escape

Immune escape is entangled with tumor recurrence, metastasis, and treatment failure [144]. Tumor cells have evolved varied kinds of mechanisms to gain immune escape abilities. One of the mechanisms is to change the components of the tumor microenvironment to affect the related immune cell functions [145]. GBM and its surrounding antigen-presenting cells express indoleamine 2,3-dioxygenase (IDO1). Tryptophan could be catabolized by IDO1 to generate kynurenine. Tryptophan-2,3-dioxygenase is expressed exclusively by high-grade gliomas and is of the same function as IDO1. Kynurenine could bind to the aryl hydrocarbon receptors of T cells, resulting in T cell antitumor response suppression [146,147]. The intermediates of kynurenine not only inhibit the proliferation of T and B cells but also induced adaptive immune attenuation [148,149,150,151]. Not limited to that, these metabolites in the kynurenine pathway also bring about DNA damage tolerance, genomic instability, and redox state alteration in glioma cells. All these events contribute to glioma immune escape [152]. Additionally, activation of the kynurenine pathway in gliomas exhausts tryptophan in the microenvironment [153]. The lack of this essential AA affects the survival of many immune and immune-related cells.

It was estimated that glioma-associated macrophages (GAMs) accounted for 30% of the GBM mass [154]. Living cells secrete single-membrane organelles resembling the same topology as the intact cells. When the vesicle-like structures form, many bioactive macromolecules and small molecular metabolites are enclosed. These secreted vesicles are called exosomes [155]. Affected by the GBM-derived exosomes, GAMs secrete exosomes with highly expressed arginase-1, an enzyme converting arginine to ornithine and urea. These GAMs-derived exosomes conferred GBM 3-to 10-fold increased resistance to TMZ [156]. Arginase-1 depletes the arginine in the tumor microenvironment. The depletion results in the proliferation arrest of the T and natural killer cells, contributing to GBM immune escape [35]. A recent glioma immune escape gene signature analysis for mice reconfirmed the roles of IDO1 and arginase-1 [157]. Gliomas with IDH mutations had fewer GAMs and were relatively sensitive to chemotherapy. The better prognosis was partially due to the weak immune escape abilities of the tumors [158]. Collectively, both the glioma cells and their secreted exosomes could shape the tumor microenvironment and contribute to tumor immune escape [159].

## 8. AAs as Diagnosis and Prognosis Biomarkers

A biomarker is valuable for both diagnosis and prognosis. Traditionally, most biomarkers were bio-macromolecules such as proteins, mutated genes, and different kinds of RNAs. Recently, small molecular metabolite biomarkers were introduced to help disease diagnosis and prognosis. Since the advent of metabolomics, many metabolite markers were readily discovered from different biological samples [160]. Metabolomics aims at quantifying as many metabolites as possible in a given system. The whole metabolites in an organism made up the organism’s metabolome. The configuration of the metabolome is phenotype-specific [161]. Nuclear magnetic resonance (NMR) and mass spectrometry (MS) are the most widely used analytical techniques [160,162]. Metabolomics analysis has gained broad applications in exploring biomarkers of glioma. A MS-based metabolomics analysis revealed that plasma arginine, glutamate, glutamine, glycine, and histidine were decreased in GBM patients. Using the 5-AA combination panel could realize a satisfied GBM diagnosis. Not limited to that, the decreased plasma leucine and phenylalanine were closely linked to a genetic deficiency in GBM [163]. Compared to the wild-type IDH1, its mutant counterpart caused decreased plasma glutamate in glioma patients [164]. Using cell lines of higher (HGG) and lower grades (LGG), an NMR-based metabolomics analysis found that 17 intracellular metabolites could be utilized as markers to separate HGG from LGG. Among the 17 metabolites, 11 were AAs [165]. Decreased taurine and increased glutamine in the HGG contribute to the separation most importantly. GSH could help to define the clinical stages of brain tumors. They were usually of lower levels in the HGG patients [166,167]. For the differential diagnosis of GBM from oligodendrogliomas, serum concentrations of cysteine were proven to be valuable [168]. α-aminoadipate is a lysine catabolic product, and it was found to be linked to a poor prognosis [169]. The elevated plasma arginine and methionine were positive indicators to predict the 2-year survival rate. On the contrary, elevated plasma kynurenate was a negative predictor [170]. AAs could also be applied to predict glioma recurrence and chemoresistance [171]. Notably, most of the AA biomarkers are involved in specific pathological processes as described in different sections of this review. Owing to the fact that metabolites are sensitive to environmental stimuli [19,172,173], metabolite biomarkers would be not reliable enough. For example, using the same MS-based strategy [164], another metabolomics analysis found opposite changes of plasma arginine and proline [174], when it was compared to the study of Nagashima H. et al. [164]. Additionally, from the technical point of view, a biomarker is expected to be of increased concentrations in a disease condition. Thus, the mentioned AA markers with decreased levels seem to be unfavorable.

According to the specific appetite for certain AAs, relevant imaging techniques have been developed. Positron emission tomography (PET) based on isotope-labeled AAs has a promising future for brain tumor diagnosis [172]. The uptake rate of the radiolabeled AAs is very low for the normal brain tissues but is very high for the gliomas. This variance created a high contrast image for the tumors against their backgrounds and even could guide biopsy [172]. The commonly used labeled AAs for PET include [11C-methyl]-L- Methionine, O-(2-[18F]-fluoroethyl)-L-tyrosine, and 3, 4-dihydroxy-6-[18F]-fluoro-L-phenylalanine [175,176,177,178]. Kinetic analysis of the labeled AA uptake rates could predict malignant transformation and prognosis [179,180,181,182,183]. For instance, a large calculated biological tumor volume indicates a poor prognosis [184,185,186] and a decreased survival potential for LGG patients [187,188,189].

## 9. Conclusions

Dysregulated AA metabolism is not unique to gliomas. Many tumors share similar rewired AA metabolism traits. Although tumorigenesis is thought to be driven by genetic mutations, only a small number of driver genes have been identified [190]. Many tumors carry zero mutations and not all the mutations found in tumors must necessarily bring about cancer. Introducing nuclei from the cancer cells to the normal cells does not transfer the malignant phenotype [191]. These scenarios raised the problem of tumorigenesis initiation. Between genetic mutations and metabolic abnormalities, which one is the driving factor? Accumulative evidence suggests that cancer might be a metabolic disease [192]. Not all tumors carry genetic mutations, but all the tumors showed metabolic abnormalities [191,192,193]. Thus, any measures that could reverse the dysregulated metabolism of the malignant cells might be an option for treating cancers. A better understanding of the rewired metabolism could improve treatment strategies.
